# An integrated pipeline for prediction of *Clostridioides difficile* infection

**DOI:** 10.1038/s41598-023-41753-7

**Published:** 2023-10-02

**Authors:** Jiang Li, Durgesh Chaudhary, Vaibhav Sharma, Vishakha Sharma, Venkatesh Avula, Paddy Ssentongo, Donna M. Wolk, Ramin Zand, Vida Abedi

**Affiliations:** 1 Department of Molecular and Functional Genomics, Geisinger Health System, Danville, PA USA; 2 Neuroscience Institute, Geisinger Health System, Danville, PA USA; 3 Geisinger Commonwealth School of Medicine, Danville, PA USA; 4 College of Osteopathic Medicine, Kansas City University, Kansas City, MO USA; 5 Department of Public Health Sciences, College of Medicine, The Pennsylvania State University, Hershey, PA USA; 6 Molecular and Microbial Diagnostics and Development, Geisinger Medical Center, Danville, PA USA; 7 Department of Neurology, College of Medicine, The Pennsylvania State University, Hershey, PA 17033 USA

**Keywords:** Computational biology and bioinformatics, Genetics, Risk factors

## Abstract

With the expansion of electronic health records(EHR)-linked genomic data comes the development of machine learning-enable models. There is a pressing need to develop robust pipelines to evaluate the performance of integrated models and minimize systemic bias. We developed a prediction model of symptomatic *Clostridioides difficile *infection(CDI) by integrating common EHR-based and genetic risk factors(rs2227306/IL8). Our pipeline includes (1) leveraging phenotyping algorithm to minimize temporal bias, (2) performing simulation studies to determine the predictive power in samples without genetic information, (3) propensity score matching to control for the confoundings, (4) selecting machine learning algorithms to capture complex feature interactions, (5) performing oversampling to address data imbalance, and (6) optimizing models and ensuring proper bias-variance trade-off. We evaluate the performance of prediction models of CDI when including common clinical risk factors and the benefit of incorporating genetic feature(s) into the models. We emphasize the importance of building a robust integrated pipeline to avoid systemic bias and thoroughly evaluating genetic features when integrated into the prediction models in the general population and subgroups.

## Introduction

With biobank and genetic data integrated with electronic health records (EHR) comes the development of predictive models designed for healthcare applications. There is an urgent need to develop robust modeling pipelines using machine learning (ML) to determine whether EHR-derived common clinical risk factors can predict the phenotype of interest and whether adding genetic factors can improve model performance. Several factors have to be considered during the model development, including (1) selection bias for the biobank data; (2) case–control imbalance; (3) temporal bias in feature acquisition; (4) impact of confounding factors; (5) optimal model selection to capture multi-way interactions; and (6) predictive power and generalizability of the final models. Here we choose *Clostridioides difficile* infection (CDI) as a proof-of-concept given its complexity and clinical importance. The purpose of this study is to develop an integrated pipeline for predicting symptomatic CDI using common EHR-derived clinical and genetic risk factors. Focusing on symptomatic CDI is driven by the fact that testing and treatment for CDI are not recommended in asymptomatic individuals^1^.

CDI is considered the most common cause of healthcare-associated diarrhea and is listed as one of the top five urgent antimicrobial resistance threats by the Centers for Disease Control and Prevention (https://www.cdc.gov/drugresistance/biggest-threats.html). Existing literature reporting CDI prediction focuses on three outcomes—symptomatic infection, severity of the infection, and recurrence. The prediction models developed in this field vary by setting, patient recruitment, data source, study design, feature selection, and algorithms. EHR-based studies have become popular due to their improved predictability, specificity, and generalizability. Host genetic susceptibility to CDI and epidemiology of *C. difficile* strains have been topics of investigations^2,3^. Intestinal inflammatory cytokines correlate more closely to disease severity than pathogen burden^4^. The same mechanism (inflammatory cytokines) applies to the inflammatory cytokine signature (plasma level of IL-6, IL-8, and TNF-α) for the prediction of COVID-19 severity and survival^5^. A previous candidate gene approach revealed that genetic polymorphisms, rs4073(–251T>A) or rs2227306(+ 781T/C) from a pro-inflammatory cytokine, IL-8, are associated with IL-8 production and predisposition to CDI^6–8^ with functional impact (eFigure 1). Genetic markers are not yet included in any established disease scoring system or clinical decision tool for risk stratification of CDI due to unclear causality, small effect size, complex gene by environment (GxE) interaction, and data availability/accessibility.

## Materials and methods

**Figure 1:**
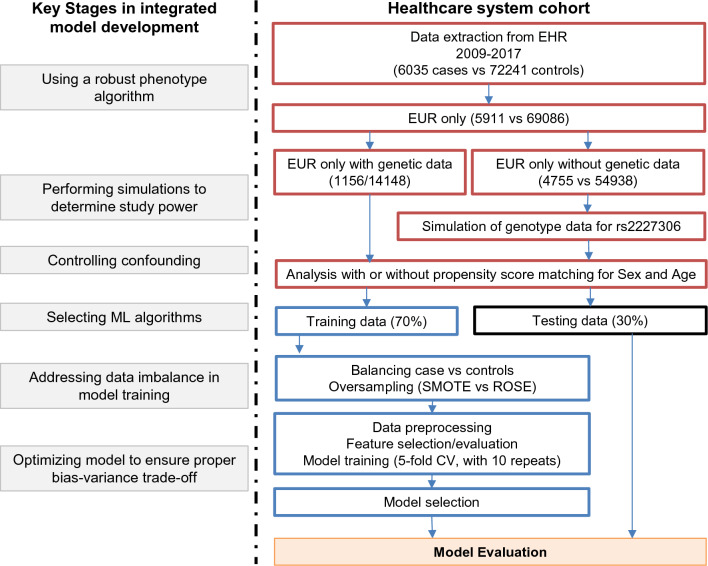
A flowchart illustrated the sample size and the pipeline for the prediction model development.

The study was conducted and reported according to the transparent reporting of a multivariable prediction model for individual prognosis or diagnosis (TRIPOD) guideline^9^. The Geisinger Institutional Review Board approved this study to meet “Non-human subject research” using de-identified information. All research was performed in accordance with relevant guidelines/regulations. Geisinger built and performs regular updates to the de-identified structured EHR database for research linked to the MyCode Community Health Initiative biorepository^10–12^. The structured EHR and matching genetics data allowed us to conduct a retrospective study on primary CDI^3^. Informed consent was obtained from all subjects and/or their legal guardian(s) for the MyCode patients. The analysis pipeline, as well as CDI-specific study parameters, are illustrated in Fig. 1.

### Robust phenotyping algorithm

The phenotype algorithm used to identify CDI cases and controls from EHR data was developed by eMERGE entitled “Phenotype Algorithm Pseudo Code (August 16, 2012)” and collected at PhenoKB (https://phekb.org/phenotype/clostridium-difficile-colitis). This algorithm adopted the golden standard, which used laboratory test data to identify CDI cases. Based on clinical symptoms, we first identified adults (age ≥ 18) with CDI from the Geisinger EHR. Three or more consecutive liquid stools within a day may be tested for *C. difficile* based on recommended guidelines^13,14^. The polymerase chain reaction was used as the laboratory reference standard test^3^. Patients tested for *C. difficile* but with negative results or exposed to antibiotics with high or moderate risk (Appendix-1) served as controls. Nongenetic risk factors collected in this study included index age, people in healthcare settings, antibiotic treatment, underlying comorbidities such as inflammatory bowel disease (IBD), type 2 diabetic mellitus (T2DM), HIV, cancer, medications such as chemo, transplant, corticosteroid, anti-TNF, proton pump inhibitors(PPI). The observation window for each risk factor was empirically defined to avoid an uneven sampling of the disease trajectory, which might lead to temporal bias in feature selection^15^. Clinical risk factors and demographic information were extracted from the structured EHR based on the *International Classification of Disease (ICD)*-related codes and medication codes (Appendix 1).

Using data from January 1, 2009, through December 31, 2017, we identified 6,035 cases and 72,241 controls. Of these, 5911 cases and 69,086 controls had self-reported European ancestry. Overall, 22.4% (1156/14,148 for case/control) of European (EUR) patients enrolled in the MyCode project with genetic data available.

### Data pre-processing

The entire cohort of participants with EUR ancestry (n = 5911/69,086) was first split based on the availability of genetic data. No missing data was observed in any of the included clinical variables. The MyCode samples were genotyped as previously reported^3^. Both SNPs (rs2227306 and rs4076) from IL-8 passed the quality control without missingness. The genetic variable is routinely treated in a dosage manner (0, 1, 2). Data extraction and pre-processing details (z-scored index age, binary codes for other variables) have previously been described^3^.

**Figure 2:**
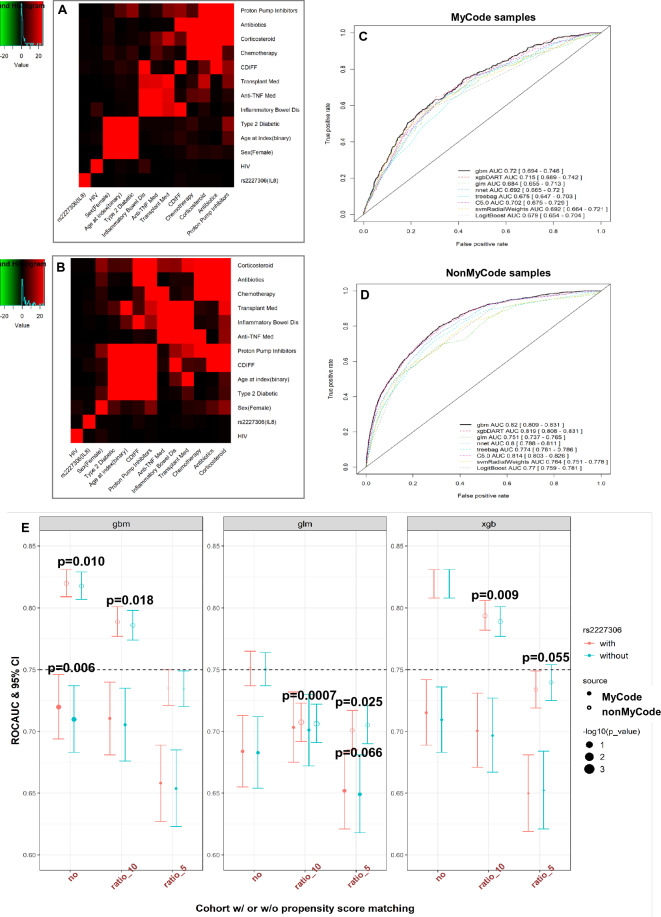
Association among features and performance of models in prediction of CDI in MyCode and nonMyCode samples with (simulated) genotypes included. (**a**, **b**) Heatmaps to show the significant association between variables employed in the prediction model using the training dataset. Data extraction and pre-processing details (z-scored index age, binary codes for other variables) have previously been described. Association among variables (index age further dummy coded) was assessed using a bivariate χ^2^ test. (**c**, **d**) To examine the discrimination power of each modeling algorithm in the testing dataset, we estimated the AUROC using common clinical risk factors for CDI with or without rs2227306 as predictors. Here the genotypes of rs2227306 were simulated in the nonMyCode samples. (**e**) The summary of AUROCs of the optimal modeling algorithms (gbm and xgbDART) versus glm using simulated rs2227306 genotype. P values represent the result of the DeLong test to compare AUROC between models with or without (simulated) genetic data included, with or without PSM for index age and sex.

Either the χ^2^ test, substituted by Fisher exact test for frequency per group ≤ 5% or the ANOVA test was performed to screen for the bivariate association. A heatmap was created to show the significance of this bivariate association based on the log-transformed p-value from each bivariate χ^2^ test (Fig. 2).

### Simulations to determine predictive power

A genotype simulation study was conducted to determine different modeling algorithms' predictive power and generalizability in the nonMyCode cohort. The genotypes of rs2227306 (IL-8) in this cohort of 4755 cases and 54,938 controls were created by a simulation strategy based on an assumption of a binomial probability distribution of each allele equaling to the prior parameter, MAF, estimated from the corresponding MyCode subgroups stratified by CDI, age, and sex (Table 2).

$${AlleleDosage}_{i \in R}=rbinom\left({N}_{i\in R},1, {MAF}_{i \in R}\right)+rbinom\left({N}_{i \in R},1, {MAF}_{i \in R}\right),$$

where R is a vector or a matrix of the summary statistics derived from the MyCode cohort, *N* and *MAF* represent the number of subjects and the corresponding MAF for each subcategory. This simulation strategy considers confounding factors such as age and sex, which may impact the association between the genetic variant and the outcome variable.

### Individual SNP association testing

Genotype and phenotype association was conducted using Logistic Regression after controlling covariates such as age or sex in subgroups stratified by sex or age (binary).

### Controlling confounding

Age and sex were identified as confounding factors for the association between genetic/nongenetic risk factors and CDI (see “Result”). They were selected as covariates in a Logistic Regression model to create propensity scores(R MatchIt package). We chose “nearest neighbor matching without replacement” to create a more balanced case:control ratio at 1:5 or 1:10, as shown in eFigure 2.

### Selecting machine learning (ML) algorithms

Model development and optimization were based on selecting the optimal sampling approach followed by a comparison of the eight classification algorithms, including Logistic Regression (glm), Gradient Boosted Classification (gbm), Extreme Gradient Boosting with the dropout regularization for regression trees (xgbDART), Bagging for tree (treebag), Neural Network [nnet, using 1-layer fully connected neural networks (shallow)], C5.0 (c5), LogitBoost (lb), and Support Vector Machine (SVM). We selected these models based on model/algorithmic diversity, performance on similar tasks, model interpretability, and ease of implementation^16^. Specifically, these set of algorithms are well-established with good performance for tabular data in a wide range of classification tasks^16^. Furthermore, Ensemble methods^17,18^ (C5.0, xgbDART, Bagging, LogitBoost) that combine multiple weak learners to create a stronger model, can lead to improved predictive performance and better generalizability. Finally, by creating decision boundaries that are complex and not linear, Neural Network and SVM are capable of capturing non-linear relationships between the variables.

### Evaluation metrics

We assessed the performance of the multivariable model in the prediction of CDI mainly by sensitivity, specificity, Positive Predictive Value (PPV), Negative Predictive Value (NPV), precision, recall, F1 score, Matthews Correlation Coefficient (MCC), accuracy, and Area Under the curve (AUROC) for a classification problem.

$${\text{F1 Score }} = { 2}*\left( {{\text{Recall }}*{\text{ Precision}}} \right) \, / \, \left( {{\text{Recall }} + {\text{ Precision}}} \right)$$

$${\text{MCC }} = \, \left( {{\text{TP}}*{\text{TN }}{-}{\text{ FP}}*{\text{FN}}} \right)/{\text{sqrt}}\left( {\left( {{\text{TP}} + {\text{FP}}} \right)\left( {{\text{TP}} + {\text{FN}}} \right)\left( {{\text{TN}} + {\text{FP}}} \right)\left( {{\text{TN}} + {\text{FN}}} \right)} \right)$$

$${\text{Accuracy }} = \, \left( {{\text{TP }} + {\text{ TN}}} \right)/\left( {{\text{TP }} + {\text{ TN }} + {\text{ FP }} + {\text{ FN}}} \right)$$

where TP = True positives; TN = True negatives; FP = False positives; FN = False negatives. Recall = TP/(TP + FN); Precision = TP/(TP + FP). Both F1 Score and MCC are calculated based on the confusion matrix and are reliable metrics for evaluating binary classification models, particularly in imbalanced datasets. F1 score combines precision and recall to provide a balanced evaluation of a model’s accuracy. MCC considers the overall balance between the four elements of the confusion matrix. The value of MCC ranges from − 1 to 1, where 1 or − 1 indicates the complete agreement or disagreement between predicted classes and actual classes, respectively, and 0 indicates completely random guessing. The result of these metrics for the training and testing datasets across eight ML algorithms was summarized in eTable3. AUROC is a graphical representation of the model’s TP rate (sensitivity/recall) versus the FP rate (1-specificity) at various probability thresholds. AUROC is insensitive to the class distribution and is often preferred over accuracy for imbalanced datasets.

### Addressing data imbalance

The imbalance of a case:control dataset can lead to biased model training, where the ML models tends to favor the majority class and underestimate the minority class, resulting in high accuracy by always predicting the majority class. The oversampled data improve ML models for the prediction of minority class when there is a significant unbalanced case–control cohort, as shown in this study (~ 1:12). We performed oversampling (using Synthetic Minority Oversampling Technique, SMOTE, and random oversampling, ROSE) of the minority class during the model training to address the class imbalance and used F1 score and MCC to assess the model performance.

The SMOTE function oversampled the minority class (rare events) using bootstrapping (perc.over = 100) and k-nearest neighbor (k = 5) to synthetically create additional observations of that event and undersampling the majority class (perc.under = 200). For each case in the original dataset belonging to the minority class, perc.over/100 new examples of that class were created. ROSE applied smoothed bootstrapping to draw artificial samples from the feature space around the minority class without undersampling the majority class. We oversampled the minority class to reach a case:control ratio of 1:1 in a training dataset.

### Model optimization

Both MyCode and nonMyCode samples were split into training and holdout data(testing) with 7:3 ratio at the beginning of this study. All the models were tuned in training data and tested in holdout data(testing data), as described in Fig. 1. The fivefold repeated CV has been applied to the corresponding training data from either MyCode or nonMyCode cohorts. The nonMyCode samples were considered as an additional dataset to determine the overfitting. However, the genotypes for nonMyCode samples were not available, which is very common for the non-biobank population. That is why we impute the genotype data for nonMyCode samples by simulation to determine the generalizability of the optimal models. A hyperparameter tuning grid was used to train the model with five-fold repeated cross-validation (CV) and ten repeats (R caret package). Model tuning was performed by an automatic grid search for each algorithm parameter randomly (eTable1 for the tuning parameters used in each final model). Finally, the testing set was used to calculate the model AUROC.

We also compared optimal models with the benchmark algorithm glm^19,20^, and extracted the feature importance of the 12 included variables. The ranks of each variable in feature importance, particularly the genetic variable, were compared across the algorithms. The DeLong test was used to compare AUROCs from two modeling algorithms and compare AUROCs of the best model with or without the genetic feature included^21,22^. For each model, the AUROC was calculated first, and the 95% confidence interval (95% CI) of AUROC was also computed. The covariance matrix $Cov\left(\widehat{A}, \widehat{B}\right)$, where $\widehat{A}$ and $\widehat{B}$ were the estimated AUROC for models A and B, respectively, of the AUROCs between the two models was calculated. The formula for the covariance between two sample proportions, $\widehat{A}, and \widehat{B}$, was given by: $Cov\left(\widehat{A}, \widehat{B}\right)$ = $\frac{(1-\widehat{A})\widehat{A}}{{n}_{a}}+ \frac{(1-\widehat{B})\widehat{B}}{{n}_{b}}+ \frac{\widehat{B}\widehat{A}}{{n}_{b}{n}_{a}} (\frac{\widehat{A}}{{n}_{a}}+ \frac{\widehat{B}}{{n}_{b}}-1)$, where ${n}_{a} and {n}_{b}$ were the sample sizes for models A and B, respectively. The *Z* statistic ($\frac{\widehat{A}-\widehat{B}}{SE(\widehat{A}-\widehat{B})}$), where $SE(\widehat{A}-\widehat{B})= \sqrt{Cov(\widehat{A},\widehat{B}})$, and p-value were computed under an assumption of normal distribution. This p-value represented the probability of observing a difference in AUROC as extreme as the one observed in the data, assuming the null hypothesis where there was no difference between the two models.

## Results

### Patients demographics

The entire dataset was split based on the availability of the genetic data. Demographic and clinical information for MyCode and nonMyCode cohorts are listed in Table 1. The case:control ratio in MyCode(n = 1156/14,148, 1:11.55) was comparable to that in the nonMyCode cohort(n = 4755/54,938, 1:12.24). This ratio (~ 1:12) indicated approximately a ten-fold enrichment for controls as shown in the previously reported EHR-based large populational studies(more than 1:100). Several demographic features (e.g., sex, age) and known clinical risk factors showed significant differences between case and control groups in MyCode and nonMyCode cohort. Their bi-variate association among all predictive variables is illustrated in Fig. 2a and b. Antibiotics were the most significant risk factor for CDI.

**Table 1:** Demographic, potential risk factors, and genotype data for MyCode and nonMyCode participants with or without propensity score matching (PSM).

Data source		NonMyCode sample (simulated genotype)							MyCode sample (original)						
Input features		w/ CDIFF	No matching		1:5 ratio matching		1:10 ratio matching		w/ CDIFF	No matching		1:5 ratio matching		1:10 ratio matching	
			w/o CDIFF	P_value*	w/o CDIFF	P_value*	w/o CDIFF	P_value*		w/o CDIFF	P_value*	w/o CDIFF	P_value	w/o CDIFF	P_value*
Sample size		4755	54,938		23,775		47,550		1156	14,148		5780		11,560	
Age at index		57.9 (23.9)	45.5 (26.9)	2.01E−229	58.2 (23.9)	0.484	50.6 (25.2)	9.31E−85	58.9 (18.0)	56.6 (19.5)	7.18E−05	59.0 (17.8)	0.836	59.1 (18.2)	0.711
Sex	Female	2763 (0.581)	30,106 (0.548)	1.23E−05	0.560	0.005	0.507	2.20E−16	677 (0.586)	8956 (0.633)	1.51E−03	3404 (0.589)	0.810	6913 (0.598)	0.410
rs2227306	CC	1569 (0.330)	18,689 (0.340)	0.100	8017 (0.337)	0.1481	16,126 (0.339)	0.1165	360 (0.311)	4952 (0.35)	0.024	2029 (0.351)	0.029	4058 (0.351)	0.023
	TC	2321 (0.488)	26,875 (0.489)		11,707 (0.492)		23,309 (0.490)		583 (0.504)	6692 (0.473)		2722 (0.471)		5456 (0.472)	
	TT	865 (0.182)	9374 (0.171)		4051 (0.170)		8115 (0.170)		213 (0.185)	2504 (0.177)		1035 (0.179)		2058 (0.178)	
Antibiotics		2007 (0.422)	8131 (0.148)	0	3590 (0.151)	2.20E−16	7085 (0.149)	2.20E−16	587 (0.508)	3169 (0.224)	2.74E−103	1277 (0.221)	1.40E−89	2589 (0.224)	2.95E−100
Inflammatory bowel dis		334 (0.070)	582 (0.011)	5.74E−225	285 (0.012)	2.20E–16	571 (0.012)	2.20E−16	110 (0.095)	279 (0.020)	2.50E−55	265 (0.046)	1.31E−11	271 (0.023)	1.53E−42
Proton pump inhibitor		1365 (0.287)	5494 (0.1)	0	2924 (0.123)	2.20E−16	5231 (0.110)	2.20E−16	465 (0.402)	3763 (0.266)	2.09E−23	1670 (0.289)	2.09E−14	3237 (0.280)	2.41E−18
Corticosteroid		1075 (0.226)	5988 (0.109)	3.45E−125	2782 (0.117)	2.20E−16	5278 (0.111)	2.20E−16	339 (0.293)	2830 (0.2)	4.91E−14	1202 (0.208)	1.58E−10	2381 (0.206)	4.17E−12
Chemotherapy		298 (0.063)	2390 (0.044)	9.68E−10	1117 (0.047)	1.21E-05	2140 (0.045)	5.24E−08	103 (0.089)	1457 (0.103)	0.1432	630 (0.109)	0.048	1202 (0.104)	0.110
Type 2 diabetic mellitus		1222 (0.257)	8351 (0.152)	1.91E−79	5635 (0.237)	0.005	8321 (0.175)	2.20E−16	412 (0.356)	4315 (0.305)	2.75E−04	1936 (0.335)	0.156	3815 (0.330)	0.069
anti-TNF medication		19 (0.004)	80 (0.001)	2.77E−04	48 (0.002)	0.001	95 (0.002)	0.000426	17 (0.015)	89 (0.006)	2.64E−03	45 (0.008)	0.022	73 (0.006)	4.62E−03
Transplant medication		126 (0.027)	808 (0.015)	7.81E−09	476 (0.020)	0.004	761 (0.016)	4.60E−07	55 (0.048)	506 (0.036)	5.03E−02	248 (0.043)	0.478	445 (0.039)	0.132
HIV		4 (0.0008)	58 (0.001)	0.817	24 (0.001)	0.748	48 (0.001)	0.6956	3 (0.003)	17 (0.001)	1.89E−01	6 (0.001)	0.179	13 (0.001)	0.179

Data were presented as the number of subjects with frequency in parentheses or Mean ± SD. All patients were subjected to a molecular test for the detection of *C. difficile*. A “*” represented statistics from the Chi-square test to determine whether there is a significant difference between the expected frequency and observed frequency in one or more categories or statistics from the ANOVA test to determine whether there is a significant difference between group means. *IBD* Inflammatory Bowel Disease, *chemo* Chemotherapy, *PPI* proton pump inhibitor medications, *steroid* Corticosteroid medications, *TNF* Anti-TNF medications, *transplant* Transplant medications, *T2DM* Type 2 Diabetes, *MHC* major histocompatibility complex, *w* with, *w/o* without.

### Genotype–phenotype association

The Logistic Regression analysis showed a significant association between rs2227306 genotype and CDI only in young MyCode patients (β = 0.138, p = 0.048 vs. β = 0.062, p = 0.263) after controlling for sex. After controlling for age, this nominal association was only observed in females (β = 0.119, p = 0.034 vs β = 0.053, p = 0.427). The minor alleles from both SNPs with the higher expression level of CXCL8 and CXCL6 were associated with an increased risk for CDI.

### Comparing oversampling methods to manage the case–control imbalance

**Figure 3:**
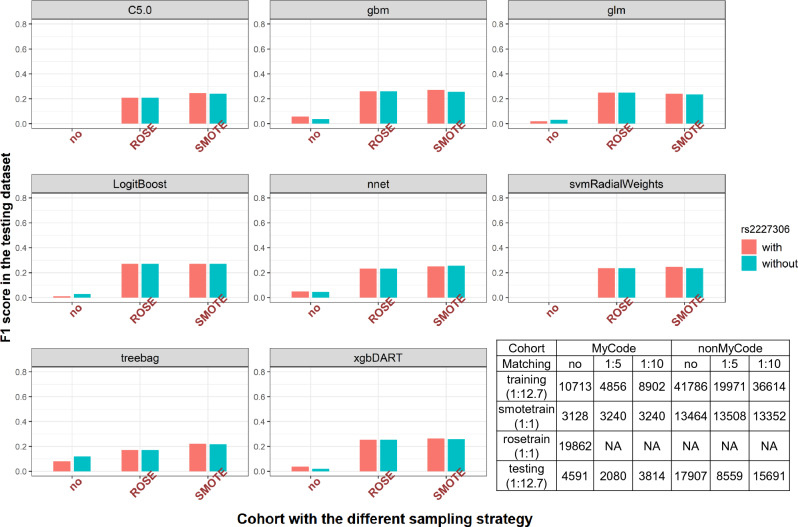
Comparing the upsampling strategies in the MyCode testing dataset using F1 score as the metric for performance. The SMOTE function oversampled the minority class (a rare event) using bootstrapping (perc.over = 100) and k-nearest neighbor (k = 5) to synthetically create additional observations of that event and undersampling the majority class (perc.under = 200). For each case in the original dataset belonging to the minority class, perc.over/100 new examples of that class will be created. The ROSE function oversampled the minority class without undersampling the majority class. Here we make the case:control ratio in the training dataset equaled to 1:1 for both oversampling strategies. F1 score, the weighted average of Precision and Recall, was selected to determine the performance of oversampling. Summary of the sample sizes for training with or without upsampling (SMOTE or ROSE) and testing dataset stratified by genetic data availability. *ROSE* upsampling cases (n = 782) to 9931 so that case:control ratio is 1:1 with controls (n = 9931) for the training dataset. *SMOTE* upsampling cases (n = 782) to 1564 so that case:control ratio is 1:1 with controls (n = 1564) for the training dataset.

SMOTE outperformed ROSE in seven out of eight examined algorithms(Fig. 3). When using xgbDART and gbm models, SMOTE in the training dataset led to better F1 (0.264 and 0.272, respectively) than the ROSE (0.253 and 0.261, respectively) in the testing dataset. The process without resampling provided the worst F1 (0.037 and 0.056, respectively). SMOTE was chosen for the following analyses. Both MCC and F1 scores showed that SMOTE yielded a slightly higher value than ROSE with or without the genetic feature included according to xgbDART and gbm models in the testing dataset (eTable 2).

### Predicting CDI in MyCode patients with or without propensity score matching (PSM)

**Figure 4:**
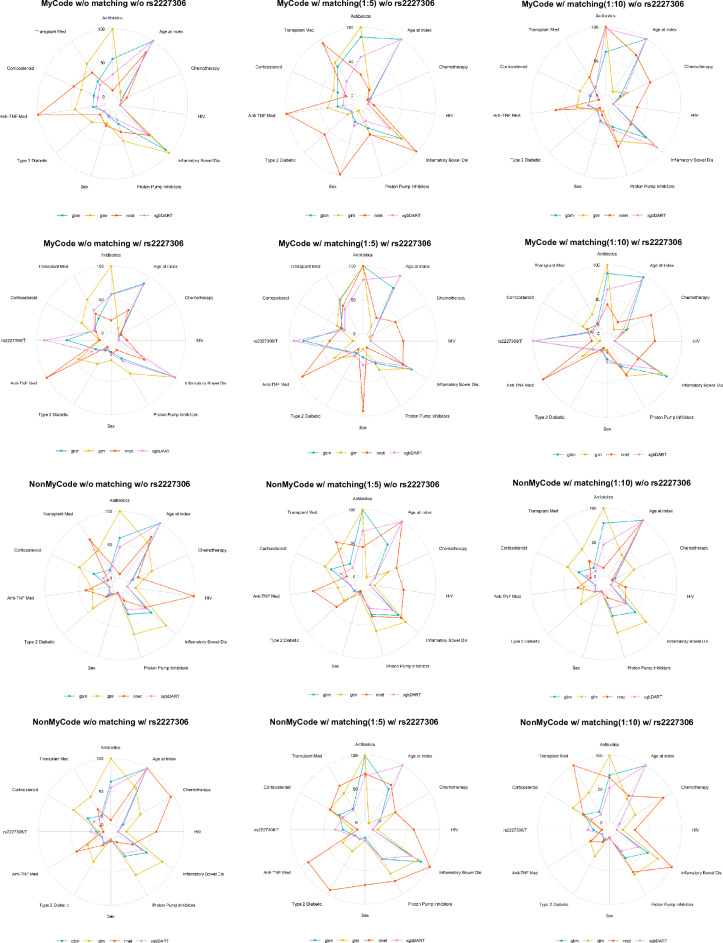
Feature importance for the cohort with or without (simulated) genetic data was plotted for two selected models (gbm and xgbDART), which outperformed other models (glm and nnet). This study was based on 12 features, including one genetic risk factor, rs2227036, from IL8. Feature importance from glm and nnet was always plotted as a control to compare the rank of the features weighted by optimal modeling algorithms (gbm and xgbDART) in MyCode (top two rows) and nonMyCode samples (bottom two rows). The genetic feature was weighted the top tier in gbm and xgbDART but not in glm and nnet irrespective of PSM in the MyCode cohort.

The best tuning parameters selected for each model based on the largest ROC value from the training dataset were summarized in eTable 1. Among eight algorithms examined, we found that using the 11 clinical risk factors with rs2227306, in conjunction with gbm and xgbDART led to superior results (with the genetic feature, AUROC_gbm_ = 0.72[0.694–0.746] versus AUROC_glm_ = 0.684[0.655–0.713], p = 1.92e−07) (Fig. 2c). There was no significant difference between gbm and xgbDART (AUROC_xgbDART_ = 0.715[0.689–0.742], p = 0.247). The genetic feature was always ranked higher than known risk factors in gbm and xgbDART compared to glm, suggesting non-parametric algorithms can better capture some nonlinear interaction(See the radar plots in Fig. 4). Compared to the base model, the integrated model provided the best discriminative power in the testing dataset, particularly for gbm (AUROC = 0.710 [0.683–0.737] vs. 0.72 [0.694–0.746], p = 0.006). xgbDART showed similar trends, better with the genetic feature, but did not reach a statistical significance (AUROC = 0.710 [0.683–0.736], vs. 0.715 [0.689–0.742], p = 0.304). Both gbm and xgbDART models provided a more balanced sensitivity (0.508 and 0.518 versus 0.481) and specificity (0.792 and 0.796 versus 0.777) and better PPV (0.178 and 0.183 versus 0.161) and NPV (0.948 and 0.949 versus 0.944) when compared to glm. No significant difference between age and sex was observed after the PSM (Table 1, eFigure 2). The algorithms with the best performance after matching (e.g. 1:10 ratio) remained gbm (AUROC_gbm_ = 0.711 [0.681–0.740]) and xgbDART (AUROC_xgbDART_ = 0.701 [0.671–0.731]) with rs2227306 (Fig. 2e). The former showed no statistically significant improvement over the model without rs2227306 (AUROC_glm_ = 0.703 [0.675–0.732]). A similar trend was observed in the matching cohort with a 1:5 ratio (Fig. 2e). Both gbm and xgbDART models ranked the genetic feature the highest in feature importance(Fig. 4 the top two rows), while glm did not. glm also ranked index age and sex lower.

### Predictive power in nonMyCode patients

The simulated genotype in the nonMyCode cohort can recapitulate the nominal association between rs2227306 and CDI (Table 2). Consistent with the result from MyCode patients, with the simulated genetic feature included, gbm (AUROC_gbm_ = 0.820 [0.809–0.831]) and xgbDART (AUROC_xgbDART_ = 0.819 [0.808–0.831]) outperformed other modeling algorithms (e.g., AUROC_glm_ = 0.751 [0.737–0.765], p = 1.68e−57) (Fig. 2d). Again, rs2227306 was ranked higher in gbm and xgbDARTthan glm (Fig. 4 the bottom two rows). The result of performance metrics (sensitivity, specificity, PPV, NPV, precision, recall, F1 score, and accuracy) for the prediction of CDI in the training and testing datasets across eight ML algorithms using SMOTE as the oversampling method was summarized in eTable3.

**Table 2:** Two simulation strategies to create the genetic data of rs2227306(IL8) in the nonMyCode cohort.

CDI (outcome)	AGE (zscore ≥ 0)	SEX (female)	Number of subjects		Minor allele frequency		χ^2^/p value	
			MyCode	NonMyCode	MyCode	NonMyCode	MyCode	NonMyCode
0	0	0	1646	12,757	0.4067	*0.4111*	7.443/0.024	*4.605/0.1*
0	0	1	4928	16,072	0.4074	*0.4098*		
0	1	0	3553	12,066	0.4191	*0.4174*		
0	1	1	4021	14,043	0.4193	*0.4233*		
1	0	0	157	527	0.4522	*0.4639*	3.515/0.173	*11.88/0.003*
1	0	1	286	688	0.4353	*0.4317*	4.701/0.095	*3.009/0.222*
1	1	0	322	1464	0.4161	*0.3924*	0.026/0.987	*7.23/0.027*
1	1	1	391	2076	0.4501	*0.4381*	3.154/0.207	*5.37/0.068*

Significant values are in italics.

This table summarizes the sample size and minor allele frequency(MAF) of each subgroup stratified by the outcome variable (CDI), age, and sex in both MyCode and nonMyCode cohorts. The genotype of rs2227306 was simulated based on an assumption of binomial distribution of each allele with frequency equaling the prior parameter (MAF) estimated from the corresponding MyCode samples. The genotype for each subject would be a combination of sampling from two binomial distributions with the same MAF.

## Discussion

We developed a prediction model of symptomatic CDI by integrating common risk factors extracted from electronic health records and genetic risk factors (rs2227306/IL8). Our modeling pipeline included steps to minimize systemic bias in the final models while adhering to best practices to improve model transparency. These steps included (1) applying robust and validated phenotyping, (2) selecting and optimizing a range of ML algorithms with a focus on attributes such as generalizability, interpretability, potential interactions, and bias-variance trade-off, (3) addressing data imbalance and performing extensive simulation studies to determine the predictive power in samples without genetic information, and finally using PSM to control for confounding factors.

Overall, our results supported that decision tree-based Ensemble methods such as gbm and xgbDART demonstrated superior discriminative power than glm(logistic regression)^16^. Both gbm and xgbDART are based on the boosting algorithm^17,18^ which is an ensemble technique that sequentially builds multiple weak learners, each focusing on the mistakes of its predecessor and assigning higher weights to misclassified instances to correct them in subsequent iterations. Ensemble methods often work better than individual ML algorithms due to the following reasons^17,18^: (1) Handing imbalanced data by giving more weight to the minority class and balancing the predictions as shown in this study; (2) Reduced Bias and Variance by combing multiple individual models(weak learners) to create a single and more robust model(strong learners); (3) Complementary learning by leveraging the complementary strengths of individual algorithms with less overfitting and improved generalization; (4) Tackling complex relationships which cannot be effectively captured by a single model; (5) Robustness to noisy data because of the averaging or voting mechanisms which often are less affected by noisy data points and outliers.

The improvements gained by including common genetic variants in the optimal models were limited and age- or sex-dependent after PSM. This finding was consistent with the decreased genetic heritability observed in late-onset compared to early-onset in multiple complex disease traits^23^, and rs2227306 (IL-8) was related to early-onset of disease^24^.

### Importance of genetic risk factor across modeling algorithms

Feature importance was considered a measure of the individual contribution of the feature for a particular classifier, regardless of the shape or direction of the feature effect. The genetic feature was consistently ranked high in the optimal models, even higher in PSM subgroups. This finding corroborates the clinical value of genetic information in prediction models. The conclusion made from the MyCode sample could partially be extended to the larger nonMyCode sample. The potential interaction between the genetic feature and clinical risk factors beyond age and sex was not recognized and implemented in the genotype simulation, which may prevent the conclusion made from the MyCode sample could fully be extended to the larger nonMyCode sample, leading to more uncertainty of the discriminative power in the model with the simulated genetic feature included. The importance of the genetic feature was ranked lowest in the Logistic Regression-based model, suggesting Logistic Regression may underestimate the contribution of the genetic variant for the prediction of CDI, highlighting the importance of capturing multi-way interactions when assessing the value of common genetic variants with a small effect size in prediction models^25^.

### Clinical relevance of the selected genetic variant from IL-8

Previous candidate gene approach of genetic predisposition of CDI has revealed a promoter polymorphism –rs4073(–251T>A) and its linkage disequilibrium SNP, rs2227306(+ 781T/C), from a pro-inflammatory cytokine, IL-8—can result in increased IL-8 production and predisposes subjects to CDI^6^, recurrent CDI^8^, or severity of CDI^7^. They are both eQTL for a gene cluster located at 4q13.3 (eFigure 1), encoding several members of the CXC chemokine family such as CXCL8 (aka IL-8), CXCL6, CXCL5, CXCL1, and more, which promote the recruitment of neutrophils to the site of infection.

Minor alleles from both SNPs are associated with an increased expression level of CXCL8 (GTEx). Increased IL-8 protein levels and CXCL5 and IL8 message levels have been associated with prolonged disease^26^. Intrarectal administration of TcdA/TcdB in a mouse model increases the expression of inflammatory mediators such as CXCL1, the murine ortholog of human IL-8^27^. PheWAS analyses from UKBIOBANK (eFigure 1) confirmed that the top phenotypes associated with rs2227306 included some laboratory variables such as “Neutrophil count”, “Neutrophil percentage”, “White blood cell count”, the latter of which contributed to a composite risk score developed in earlier studies in the prediction of CDI severity^28,29^ (Table 3). Our study is the first to evaluate the integrated model by including the genetic and common clinical risk factors using various optimized ML algorithms to predict CDI.

**Table 3:** Summary of the reported clinical decision tools to predict *Clostridioides difficile* infection (CDI), severity, or recurrence.

Study design	Study characteristics (timeline, sample size, and features)	Outcome measures	Handling missing data	Algorithms	Performance	Summary
Retrospective cohort^43^ (2021)	EHR (May 2010 to July 2014) from single healthcare; 9986 CDI cases and 2 230 354 members without CDI; 104, 518 hospital discharges for validation; case:control ≈ 1:23 ≈ 20 Risk factors	CDI	Not addressed	Univariate logistic regression Logistic Regression to develop 2 risk scores. Model 1: hospital discharge IDRSA. Model 2: random IDRSA	Model 1: using hospital discharge as the IDRSA, C-statistic of 0.848 in subsequent 31–365 days; Model 2: using a random date as the IDRSA, *C*-statistic 0.722	Identification of high-risk populations for CDiff vaccine trials to determine the study feasibility (sample size and time to completion)
Case–control study^36^ (2019)	Adult patients admitted to a multicenter study from July 1, 2015, to July 1, 2017, who received systemic antibiotics 200 subjects (100 cases and 100 controls) Reported 2 features	*CDI (hospital-associated)*	Not addressed	Univariate logistic regression Multivariate logistic regression model to formulate a point-based risk prediction model	Sensitivity and specificity were 76% and 49% Highest accuracy (63%) AUROC = 0.7	A simple-to-implement hospital-onset CDI risk model; including only independent risks that can be obtained immediately on presentation to the healthcare facility
Retrospective Cohort^42^ (2018)	EHR-based adult inpatients admitted to two healthcare systems one( January 1, 2010, and January 1, 2016) and the other (June 1, 2012, and June 1, 2014) 191,014 (155,009/36,005 for training/testing) and 65,718 (33,477/32,241 for training/testing) for two healthcare systems respectively Case:control ≈ 1:100 4836 and 1837 features from the two healthcare systems respectively	CDI	Not addressed	L2 regularized logistic regression Logistic regression to create a daily risk score for risk stratification	AUROC = 0.82 [0.80–0.84] and 0.75 [0.73–0.78] for two cohorts respectively	Many of the top predictive factors differed between the cohorts from two healthcare systems Institution-specific models instead of “one-size-fits-all” models
Retrospective Cohort^41^ (2016)	Population-based sample Medicare beneficiaries aged 65 and older on January 1, 2008, with continuous Medicare coverage from January 1, 2008, through December 31, 2009.Inpatient setting (58.5%) Of 1,165,165 Medicare beneficiaries meeting the enrollment criteria, 6,838 had an incident CDI episode; case:control = 1:170 22 features	CDI	Not addressed	Logistic regression model for feature selection sequentially removes features with < 0.8 change in C-statistic; A weighted score was developed for each of the risk factors based on its odds ratio, with the sum of all of the risk values representing a participant’s risk score	C-statistic = 0.858 NPV = 98.7%	Developed a risk stratification scoring system Emphasized the age-dependent CDI
Retrospective cohort^40^ (2016)	Admitted over a 1-year period (2013) Total of 61,482 subjects, Discovery dataset (40,990) and validation dataset (20,492) case:control ≈1:200 ~ 25 features	CDI (hospital-associated)	Not addressed	Multivariable analysis to identify risk factors individually Multivariable model based on six risk factors to develop a risk score	Sensitivity = 82.0%; Specificity = 75.7%; AUROC = 0.85	Developed a clinical prediction rule to identify patients at high risk for primary CDI
Retrospective cohort (longitudinal)^39^ (2015)	Hospital discharge data and pharmacy data from two large academic centers linked to active population-based CDI surveillance data from the Emerging Infections Program (EIP) Of the 35,186 index hospitalizations, 288 (0.82%) had CDI ≥ 28 days post discharge 39 features to begin with, 4 features left	CDI (Having CDI ≥ 28 days post discharge	Not addressed	Cox proportional hazards model (stepwise backward selection) for low and high risk groups	C-statistics = 0.75	Develop a risk score applied at discharge to identify a risk of CDI ≥ 28 days post discharge
Case–control study^35^ (2015)	Patients admitted between January 2005 and December 2011 from a single healthcare system Discovery:180 cases and 330 controls; Validation: 97 cases and 417 controls; case:control ≈ 1:120 12 features	CDI (hospital-associated)	Not addressed	Stepwise backward elimination to determine the best fit model Logistic regression to develop a simplified risk score	Corrected AUROC = 0.81[0.77–0.85]; calibration: Brier score = 0.004	Developed and validated a model to predict the incident CDI in hospitalized patients who receive systemic antibiotic treatment
Retrospective cohort^38^ (2014)	All patients admitted on or after April 12, 2011 and discharged on or before April 12 Training: 34 846 admissions (372 cases of CDI).Validation: 34 722 admissions (355 cases of CDI) Case:Control ≈1:100 14 features (EHR Model) 1017 features (Curated Model) 10,859 features ( EHR ALL)	CDI (hours from the time of admission)	Not addressed	L2-Regularized Logistic Regression 3 Models Compared based on different number of features included in the final models to discriminate low-risk from high-risk patients	AUROC Risk Period > 24 h EHR = 0.81(0.79–0.83) Curated = 0.72 (0.69–0.75) EHR ALL = 0.8140 (0.80–0.83) Risk Period > 48 h EHR = 0.7886 (0.76–0.82) Curated = 0.69 (0.66–0.72) EHR ALL = 0.79 (0.76–0.81)	Additional features from EHR data improved prediction and outperformed the model only considering a small set of known clinical risk factors
Retrospective cohort^37^ (2014)	all inpatient visits for the 2 years between April 2011 and April 2013 1348 test positive cases of C difficile out of 132 853 admissions from three hospitals, varying in size and location; case:control = 1:100 578 binary features; Different feature spaces including common (256) and specific features	CDI (hospital-associated)	Missingness has been discussed; source feature space and target feature space	L2-regularized logistic regression Multivariate Logistic regression	AUROC ≈ 0.80 varied by the approach and target task	The external data from other hospitals can be successfully and efficiently incorporated into hospital-specific models
Case–control study^34^ (2014)	Not available (abstract only) 8 Known risk factors	*CDI*	Not addressed	All feature included Multivariate regression model to create a weighted score tool	Sensitivity = 92%; Specificity = 39%	Developed a weighted scoring tool to predict incident CDI
Retrospective cohort^33^ (2014)	A consecutive cohort of patients admitted to the adult medical service over a period of 17 months (June 2011 to October 2012) 62 out of 7026 patients with over 48 h hospital stay having hospital-onset CDI cases; case:control = 1: 100 Reported 6 features	CDI (hospital-onset)	Addressed for missingness in serum albumin level	Univariate analysis to determine the potential risk factors included in the model Multivariable logistic regression model using a forward stepwise selection for features	AUROC = 0.94 [ 0.92–0.95]. Sensitivity = 98.3% [90.2–99.9]; Specificity = 85.2% [84.3–86.0]	Developed a predictive scale for hospital-onset CDI which can be used for risk stratification
Retrospective Cohort^32^ (2011)	Patients admitted for ≥ 48 h during the calendar year 2003 from a single healthcare system 35,350 total admissions & 329 CDI cases. Case:control ≈ 1:100 11 features	CDI	Not addressed	Feature selection based on high dimensional data reduction techniques such as PCA, cluster analyses Logistic stepwise regression to determine the best fit model Logistic regression also tests for some feature interactions	C index = 0.88; Brier score 0.009)	Developed and validated a CDI risk prediction model using EHR with strong discriminative capacity
Retrospective cohort^31^ (2009)	Three phases design: discovery dataset (NA), testing dataset (n = 1468), and external validation (n = 29,425)	*CDI Clostridium difficile-*associated disease (CDAD)	Not addressed	Logistic regression model	AUROC = 0.827; Sensitivity = 70% and specificity = 95%	Developed a predictive score to predict patients’ risk of developing CDAD
Retrospective cohort^30^ (2008)	Temporal split. development cohort (March 2005 to December 2006) and a validation cohort (January 2007 to October 2007) a cohort of hospital patients given broad-spectrum antibiotics 392 (288/104) out of 54,226 (41,224/13,002). Case:control ≈ 1:100 Reported 4 features	*CDI*	Not addressed	Logistic regression model to identify significant predictors individually A scoring algorithm to create four categories of CDI risk	AUROC = 0.712	Developed an easily implemented risk index for risk stratification of patients
Prospective Cohort^47^ (2018)	Patients symptomatic of CDIFF between July 2014 and February 2015 from 14 Spanish hospitals 274 (Training dataset); 183 (Validation cohort). Reported 4 features	Recurrence	Not addressed	Logistic regression Model with model calibration using Hosmer–Lemeshow test Logistic regression to form a GEIH-CDI score	AUROC = 0.72 (0.65–0.79)	Develop a risk score for recurrent CDI prediction and stratification
Retrospective cohort^50^ (2019)	First episode of adult CDI from January 1, to December 2015 For recurrence, 36 vs 191 For poor outcome, 70 vs157 (no testing dataset) ≈35 features	Recurrence Severity	Addressed	Univariate analyses; Backward stepwise multivariate logistic regression Multivariate regression	AUROC = 0.728/0.789 for clinical model; 0.775/0.801 for EIA-included model; 0.785/0.804 for PCR-including model for recurrence/severity	
Retrospective cohort (longitudinal)^46^ (2017)	EHR (2007–2013) from single healthcare Training: 9,386 incident CDI & 1,311 first CDI recurrence; testing: 1865 incident CDI &144 recurrent CDI 150 predictors	Recurrence (inpatient or outpatient)	Right-censored data	Univariate and bivariate regression Competing risk discrete survival models and Cox competing risk survival regression	Basic (*C*-statistic: 0.591, sensitivity: 75.69, specificity: 41.19). Enhanced (*C*-statistic: 0.587, sensitivity: 69.44, specificity: 43.64). Automated (*C*-statistic: 0.605, sensitivity: 79.17, specificity: 32.04). Zilberg (c-stat: 0.591, sensitivity: 74.31, specificity: 39.03)	None of the models showed a well discriminative power Suggest including environmental and ecological predictors
Retrospective Cohort^45^ (2015)	Patients with lab tests positive for CDIFF between January 2009 and June 2013 at single healthcare system 198 CDI with 30 having CDR and breaking into 70% & 30% for training and testing 25 features	Recurrence	Not addressed	All features included Random Forest	Sensitivity(83.3%), Specificity(63.1%), and AUROC (0.826)	Expecting Random Forest model with a higher performance
Retrospective Cohort^44^ (2013)	January 2006 – October 2010 from 4-hospital Heath Care Organization 198 out of 829 with relapse for 56 days of follow-up Reported 6 features	Relapse	Not addressed	Univariate logistic regression Multivariate logistic regression	Predicted 14.6% of CDI Episodes	Comprehensive EHR can be used to identify patients at high risk for CDI relapse. Major risk factors include antibiotic and PPI exposure
Retrospective Cohort^49^ (2019)	Adult inpatients diagnosed with CDI from October 2010 to January 2013 at single healthcare. 89 out of 1144 cases of CDI having complicated CDI; 894 cases for training and 224 cases for testing 23 features for the curated model; 4271 features from EHR; final selected 900 features; 923 features for EHR + curated	Severity (3Day Complications)	No imputation or case-wise deletion	Compared EHR-based model to one based on a small set of manually curated features L2 regularization regression model Logistic regression	AUROC = 0.69 [0.55–0.83) on the day of CDI diagnosis; AUROC = 0.90 [0.83–0.95] 2 days after CDI diagnosis; outperformed curated feature model with AUROC = 0.84 [0.75–0.91]	Develop a model based on EHR data to accurately stratify CDI cases according to their risk of developing complications
Prospective cohort^48^ (2015)	Discovery dataset: Boston site from December 2004 to January 2006, Validation dataset: Dublin site from November 2007 to June 2009, & Houston site from January 2006 to August 2010 251 for Discovery and 345 for validation 3 features (Age, WBC, and Creatinine)	Severity	Not addressed	Univariate logistic regression Multivariate logistic regression analysis to form a Clostridium difficile severity score (CDSS)	AUROC = 0.725 [0.675–0.769]	Developed a CDSS scoring system to predict severe CDI
Retrospective cohort^29^, et al. (2011)	January 2004 and December 2007 255 patients 4 risk factors (history of malignancy + 3 laboratory variables)	Severity	Not addressed	Univariate analysis Composite scoring: CDI severity index score	AUROC = 0.78; Sensitivity = 82%; Specificity = 65%	Develop a composite score for risk stratification of severe CDI
Prospective cohort^28^ (2009)	A single healthcare 8 out of 58 for day1 and 75 for day 3 having severe complications 3 Laboratory variables	Severity	Not addressed	No feature selection Composite scoring: RUWA scoring system	Sensitivity: 80.0% [39.4–96.3] and 62.5% [32.3–85.6]; Specificity: 77.4% [73.5–78.9] and 82.1% [78.5–84.8] on day 1 and day 3 respectively	the Ratio of white cell count on the day of the positive C. difficile toxin test to two days previously, as well as the Urea, White cell count and Albumin on the day of the positive C. difficile toxin test

We conducted a comprehensive search on PubMed and Web of Science by combining two major themes of *Clostridioides difficile* and prediction. The search strings for prediction of *Clostridioides difficile* infection were: ("clostridioides difficile "[Mesh] OR "clostridium difficile"[Mesh]) AND ("prediction"[Mesh] OR "machine learning”). We focused on human subject studies. Any review articles and studies focusing on metagenomics or microbiome data were excluded. The PubMed database was searched, and studies available between January 1, 1990, and May 31, 2021, were included. We also checked the reference from each included study, and additional studies that were missed during the initial search were appended. A total of 23 original articles were included.

### In the context of other similar studies

Since the testing and treatment are not recommended in asymptomatic carriers of *C. difficile*^1^, we direct our focus on symptomatic CDI. The results from this study may eventually facilitate at-risk patient stratification for targeted treatment in patients more likely to benefit from emerging prevention or treatment options such as a vaccine, fidaxomicin, monoclonal antibodies, and fecal microbiota transplantation. Results may also support more granular substratification for therapeutic trials.

Controls were defined as patients without CDI, based on negative molecular laboratory results or exposed to similar risk factors, such as antibiotic use. Because of using the eMERGE phenotype algorithm with the inclusion/exclusion criteria to define controls, our case:control ratio was approximately 1:10, which was a tenfold enrichment of controls with increased risk for CDI, compared to more than 1:100 ratio summarized from other retrospective cohorts studies (Table 3). This enrichment would make the prediction more challenging.

The results from a subgroup with PSM suggested that the contribution of the genetic variant in model prediction was minimum in elderly patients. This finding was consistent with the decreased genetic heritability observed in late-onset compared to early-onset in multiple complex disease traits^23^, and rs2227306 (IL-8) was related to early-onset of disease^24^.

As summarized in Table 3, the majority of the predictive models for CDI^30–36^ are not based on large EHR or claims databases until recently^37–43^, whereas studies performed on recurrence^44–47^ or severity^28,29,48,49^ include very small cohorts. Further, the existing studies do not compare algorithms; instead, they focus on the amount of information extracted necessary for improved prediction. In general, the amount of information, such as the number of variables, correlates with model performance; however, including hundreds of variables can lead to models with lower interpretability and reduced generalizability to other healthcare systems. For example, some institution-specific features can rank in the top tier in feature importance; therefore, a healthcare-based model may have limited discriminative power in the prediction of individuals from other healthcare systems when geographic, social-economic, and clinical management environments differ significantly.

The generalizability of developed prediction models from a single healthcare system to others is debatable^37^. Since this study aims to utilize only common clinical risk factors readily available in most EHRs to build a prediction model, the conclusion made from this study could have better generalizability and may be easier to implement elsewhere. For these reasons, we propose that this integrated model is more transferable to EHR than complex models with manually curated variables and datasets.

### Strength and limitation

The strength of this study lies in the following, (1) development of a prediction model of symptomatic CDI by integration of genetic and common clinical risk factors; (2) evaluation of several advanced ML algorithms to compare their performance; (3) identification of an association between the genetic variant and the outcome variable, which was confounded by age and sex; (4) determination of the value of the genetic feature in its contribution to the model performance, in general, and propensity score matched subgroups; and (5) identification of the selection bias in the cohort with genetic data available.

This study has some limitations, including (1) the accuracy of EHR data collection and recording processes that may vary by clinician, hospital, and over time to possibly prevent the generalizability of developed models to other healthcare systems; (2) our data came from a single healthcare system with a patient population that was predominantly European ancestry. The features selected from this homogenous population may not best represent or cover the complexity of feature space derived from a heterogenous population; (3) we only tested a common genetic variant with a high MAF. We expect the polygenic risk score developed from the consortium-based GWAS with individual effect size estimated from thousands of genetic variants would better represent the genetic liability to CDI and other complex diseases.

In conclusion, we showed that developing robust prediction models for CDI, and perhaps other complex conditions, requires a step-wise approach to ensure the highest level of transparency and lowest possible systemic bias. This study leveraged CDI as a disease model to demonstrate that although genetic information may improve predictions, the benefit of including genetic feature(s) in the prediction models should be thoroughly evaluated.

### Supplementary Information

Supplementary Figures.

Supplementary Tables.

Supplementary Information 3.

## Data Availability

The patient-level EHR data analyzed in this study may be shared with a third party upon execution of data sharing agreement for reasonable requests. Such requests should be addressed to V. Abedi. All the codes can be found at TheDecodeLab/Prediction_of_CDI_by_EHR_and_Genetics (https://github.com/TheDecodeLab/Prediction_of_CDI_by_EHR_and_Genetics).

## Abbreviations

AUROC: Area under the receiver operating characteristic
Treebag: Bagging for tree
BMI: Body Mass Index
CXCL: Chemokine (C–X–C motif) ligand
*C. difficile*: *Clostridioides* *difficile*
CDI: *Clostridioides** difficile* Infection
CV: Cross-validation
EHR: Electronic health records
eQTL: Expression quantitative trait loci
xgbDART: Extreme gradient boosting with the dropout regularization for regression trees
GWAS: Genome-wide association study
gbm: Gradient boosting machine
IBD: Inflammatory bowel disease
IL-8: Interleukin-8
ICD: International classification of disease
ML: Machine learning
MHC: Major histocompatibility complex
MAF: Minor allele frequency
MCC: Matthews correlation coefficient
nnet: Neural network
NPV: Negative predictive value
PheWAS: Phenome-wide association study
PPV: Positive predictive value
PPI: Proton-pump inhibitor
SVM: Support vector machine
PSM: Propensity score matching
SMOTE: Synthetic minority oversampling technique
ROSE: Random oversampling
T2DM: Type 2 diabetic mellitus

## References

[1] Khanna S, Pardi DS (2012). Clostridium difficile infection: New insights into management. Mayo Clin. Proc..

[2] Berkell M (2021). Microbiota-based markers predictive of development of *Clostridioides difficile* infection. Nat. Commun..

[3] Li J (2021). Variants at the MHC region associate with susceptibility to *Clostridioides difficile* infection: A genome-wide association study using comprehensive electronic health records. Front. Immunol..

[4] El Feghaly RE (2013). Markers of intestinal inflammation, not bacterial burden, correlate with clinical outcomes in *Clostridium difficile* infection. Clin. Infect. Dis..

[5] Del Valle DM (2020). An inflammatory cytokine signature predicts COVID-19 severity and survival. Nat. Med..

[6] Jiang ZD (2006). A common polymorphism in the interleukin 8 gene promoter is associated with *Clostridium difficile* diarrhea. Am. J. Gastroenterol..

[7] Czepiel J (2018). The presence of IL-8 +781 T/C polymorphism is associated with the parameters of severe *Clostridium difficile* infection. Microb. Pathog..

[8] Garey KW (2010). A common polymorphism in the interleukin-8 gene promoter is associated with an increased risk for recurrent *Clostridium difficile* infection. Clin. Infect. Dis..

[9] Collins GS, Reitsma JB, Altman DG, Moons KG (2015). Transparent reporting of a multivariable prediction model for individual prognosis or diagnosis (TRIPOD): The TRIPOD statement. BMC Med..

[10] Carey DJ (2016). The Geisinger MyCode community health initiative: An electronic health record-linked biobank for precision medicine research. Genet. Med..

[11] Abul-Husn NS (2016). Genetic identification of familial hypercholesterolemia within a single US health care system. Science.

[12] Dewey FE (2016). Distribution and clinical impact of functional variants in 50,726 whole-exome sequences from the DiscovEHR study. Science.

[13] Burnham CA, Carroll KC (2013). Diagnosis of *Clostridium difficile* infection: An ongoing conundrum for clinicians and for clinical laboratories. Clin. Microbiol. Rev..

[14] McDonald LC (2018). Clinical practice guidelines for *Clostridium difficile* infection in adults and children: 2017 update by the infectious diseases society of america (IDSA) and society for healthcare epidemiology of America (SHEA). Clin. Infect. Dis..

[15] Yuan W (2021). Temporal bias in case-control design: Preventing reliable predictions of the future. Nat. Commun..

[16] Borisov V (2022). Deep neural networks and tabular data: A survey. IEEE Trans. Neural Netw. Learn. Syst..

[17] Zhou, Z.-H. Ensemble methods foundations and algorithms. in *Ensemble Methods*, 23–95 (2012).

[18] Rokach, L. Chapter 3. Introduction to ensemble learning. in *Ensemble Learning Pattern Classification Using Ensemble Methods 2nd Edition*, 51–104, 10.1142/9789811201967_0003 (2019).

[19] Abedi V (2021). Prediction of long-term stroke recurrence using machine learning models. J. Clin. Med..

[20] Abedi V (2021). Predicting short and long-term mortality after acute ischemic stroke using EHR. J. Neurol. Sci..

[21] DeLong ER, DeLong DM, Clarke-Pearson DL (1988). Comparing the areas under two or more correlated receiver operating characteristic curves: A nonparametric approach. Biometrics.

[22] Sun X, Xu W (2014). Fast implementation of DeLong’s algorithm for comparing the areas under correlated receiver operating characteristic curves. IEEE Signal Process. Lett..

[23] Mars N (2020). Polygenic and clinical risk scores and their impact on age at onset and prediction of cardiometabolic diseases and common cancers. Nat. Med..

[24] Emonts M (2011). Polymorphisms in genes controlling inflammation and tissue repair in rheumatoid arthritis: A case control study. BMC Med. Genet..

[25] Xu D (2021). Quantitative disease risk scores from EHR with applications to clinical risk stratification and genetic studies. NPJ Digit. Med..

[26] El Feghaly RE, Stauber JL, Tarr PI, Haslam DB (2013). Intestinal inflammatory biomarkers and outcome in pediatric *Clostridium difficile* infections. J. Pediatr..

[27] Hirota SA (2012). Intrarectal instillation of Clostridium difficile toxin A triggers colonic inflammation and tissue damage: Development of a novel and efficient mouse model of *Clostridium difficile* toxin exposure. Infect. Immun..

[28] Drew RJ, Boyle B (2009). RUWA scoring system: A novel predictive tool for the identification of patients at high risk for complications from *Clostridium difficile* infection. J. Hosp. Infect..

[29] Lungulescu OA, Cao W, Gatskevich E, Tlhabano L, Stratidis JG (2011). CSI: A severity index for *Clostridium difficile* infection at the time of admission. J. Hosp. Infect..

[30] Garey KW (2008). A clinical risk index for *Clostridium difficile* infection in hospitalised patients receiving broad-spectrum antibiotics. J. Hosp. Infect..

[31] Tanner J, Khan D, Anthony D, Paton J (2009). Waterlow score to predict patients at risk of developing *Clostridium difficile*-associated disease. J. Hosp. Infect..

[32] Dubberke ER (2011). Development and validation of a *Clostridium difficile* infection risk prediction model. Infect. Control Hosp. Epidemiol..

[33] Chandra S, Thapa R, Marur S, Jani N (2014). Validation of a clinical prediction scale for hospital-onset *Clostridium difficile* infection. J. Clin. Gastroenterol..

[34] Smith LA (2014). Development and validation of a *Clostridium difficile* risk assessment tool. AACN Adv. Crit. Care.

[35] van Werkhoven CH (2015). Identification of patients at high risk for *Clostridium difficile* infection: Development and validation of a risk prediction model in hospitalized patients treated with antibiotics. Clin. Microbiol. Infect..

[36] Tilton CS, Johnson SW (2019). Development of a risk prediction model for hospital-onset *Clostridium difficile* infection in patients receiving systemic antibiotics. Am. J. Infect. Control.

[37] Wiens J, Guttag J, Horvitz E (2014). A study in transfer learning: Leveraging data from multiple hospitals to enhance hospital-specific predictions. J. Am. Med. Inform. Assoc..

[38] Wiens J, Campbell WN, Franklin ES, Guttag JV, Horvitz E (2014). Learning data-driven patient risk stratification models for *Clostridium difficile*. Open Forum Infect. Dis..

[39] Baggs J (2015). Identification of population at risk for future *Clostridium difficile* infection following hospital discharge to be targeted for vaccine trials. Vaccine.

[40] Press A (2016). Developing a clinical prediction rule for first hospital-onset *Clostridium difficile* infections: A retrospective observational study. Infect. Control Hosp. Epidemiol..

[41] Zilberberg MD, Shorr AF, Wang L, Baser O, Yu H (2016). Development and validation of a risk score for *Clostridium difficile* infection in medicare beneficiaries: A population-based cohort study. J. Am. Geriatr. Soc..

[42] Oh J (2018). A generalizable, data-driven approach to predict daily risk of *Clostridium difficile* infection at two large academic health centers. Infect. Control Hosp. Epidemiol..

[43] Aukes L (2021). A risk score to predict clostridioides difficile infection. Open Forum Infect. Dis..

[44] Hebert C, Du H, Peterson LR, Robicsek A (2013). Electronic health record-based detection of risk factors for *Clostridium difficile* infection relapse. Infect. Control Hosp. Epidemiol..

[45] LaBarbera FD, Nikiforov I, Parvathenani A, Pramil V, Gorrepati S (2015). A prediction model for *Clostridium difficile* recurrence. J. Community Hosp. Intern. Med. Perspect..

[46] Escobar GJ (2017). Prediction of recurrent *Clostridium difficile* infection using comprehensive electronic medical records in an integrated healthcare delivery system. Infect. Control Hosp. Epidemiol..

[47] Cobo J (2018). Prediction of recurrent clostridium difficile infection at the bedside: The GEIH-CDI score. Int. J. Antimicrob. Agents.

[48] Na X (2015). A multi-center prospective derivation and validation of a clinical prediction tool for severe *Clostridium difficile* infection. PLoS ONE.

[49] Li BY, Oh J, Young VB, Rao K, Wiens J (2019). Using machine learning and the electronic health record to predict complicated *Clostridium difficile* infection. Open Forum Infect. Dis..

[50] Origuen J (2019). Toxin B PCR amplification cycle threshold adds little to clinical variables for predicting outcomes in *Clostridium difficile* infection: A retrospective cohort study. J. Clin. Microbiol..

