# Supplementary material for: An integrated pipeline for prediction of Clostridioides difficile infection
Source: Sci Rep. 2023 Oct 2;13:16532. doi: 10.1038/s41598-023-41753-7 (PMC10545794; doi:10.1038/s41598-023-41753-7)

eFigure1. Functional annotation of rs2227306 and PheWAS results reported from UKBiobank data.
a. eQTL data V7 release was downloaded from GTEx Portal (https://gtexportal.org/home/); pQTL data generated from human plasma proteome was downloaded from Somalogic Box. **b**. Open Targets Genetics (www.OpenTargets.org) for PheWAS from UKbiobank and other publications.

a

Gene	Overall V2G	Distance (Canonical TSS)	pQTL (Sun, 2018)	eQTL	Enhancer-TSS corr (FANTOM5)	DHS-promoter corr (Thurman, 2012)	PCHi-C (Jung, 2019)	VEP (Ensembl)
CXCL8	0.281	797		0.9				intron_variant
PF4V1	0.272	111958	0.1	1				
CXCL6	0.190	95302		0.6				
CXCL5	0.154	257339		0.6			0.1	
CXCL1	0.145	128055		0.4				
UMLILO	0.091	31036						
RASSF6	0.073	120707						
PF4	0.054	240786						
PPBP	0.054	246852						
LINC02499	0.054	232535						
CXCL3	0.045	297469						
AFM	0.045	259593						
AFP	0.036	310200						
ALB	0.036	344224						
MTHFD2L	0.027	372836						
CXCL2	0.027	357858						
AC093677.2	0.018	417035						
ANKRD17	0.009	482540						
AC053527.1	0.009	482129						

V2G score was calculated for the purpose of fine-mapping. V2G = assigned variant to gene score based on Variant-to-Gene(V2G) pipeline (genetics-docs.opentargets.org) of four main data types (QTLs, chromatin interaction experiments, VEP, and TSS).

b

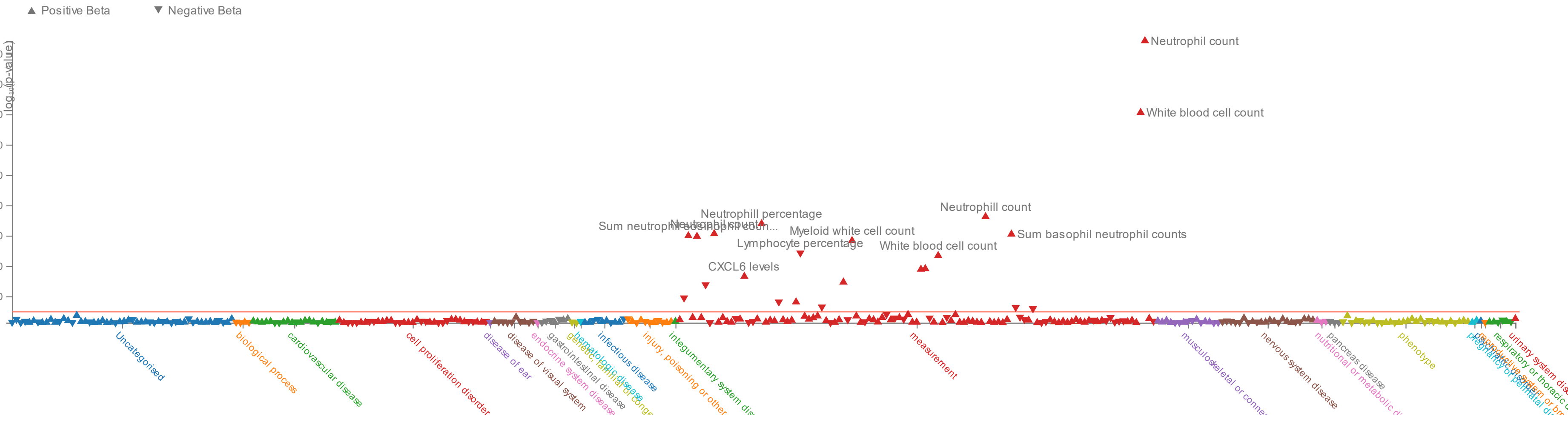

eFigure2. LOVE plot to display the improvement in standardized mean differences after propensity score matching for index age and sex with case:control ratio controlled at 1:5 or 1:10. Age and sex were identified as confounding factors for the association between genetic/nongenetic risk factors and CDI (see result section). They were selected as covariates in a Logistic Regression model to create propensity scores (R MatchIt package). We chose “nearest neighbor matching without replacement” to create a more balanced case:control ratio. The love plot (R cobalt package) demonstrated how well the improvement of standardized mean differences deviated from zero for each covariate before and after PSM in MyCode (left) and nonMyCode (right) samples after PSM at 1:5 (upper row) or 1:10 (bottom row).

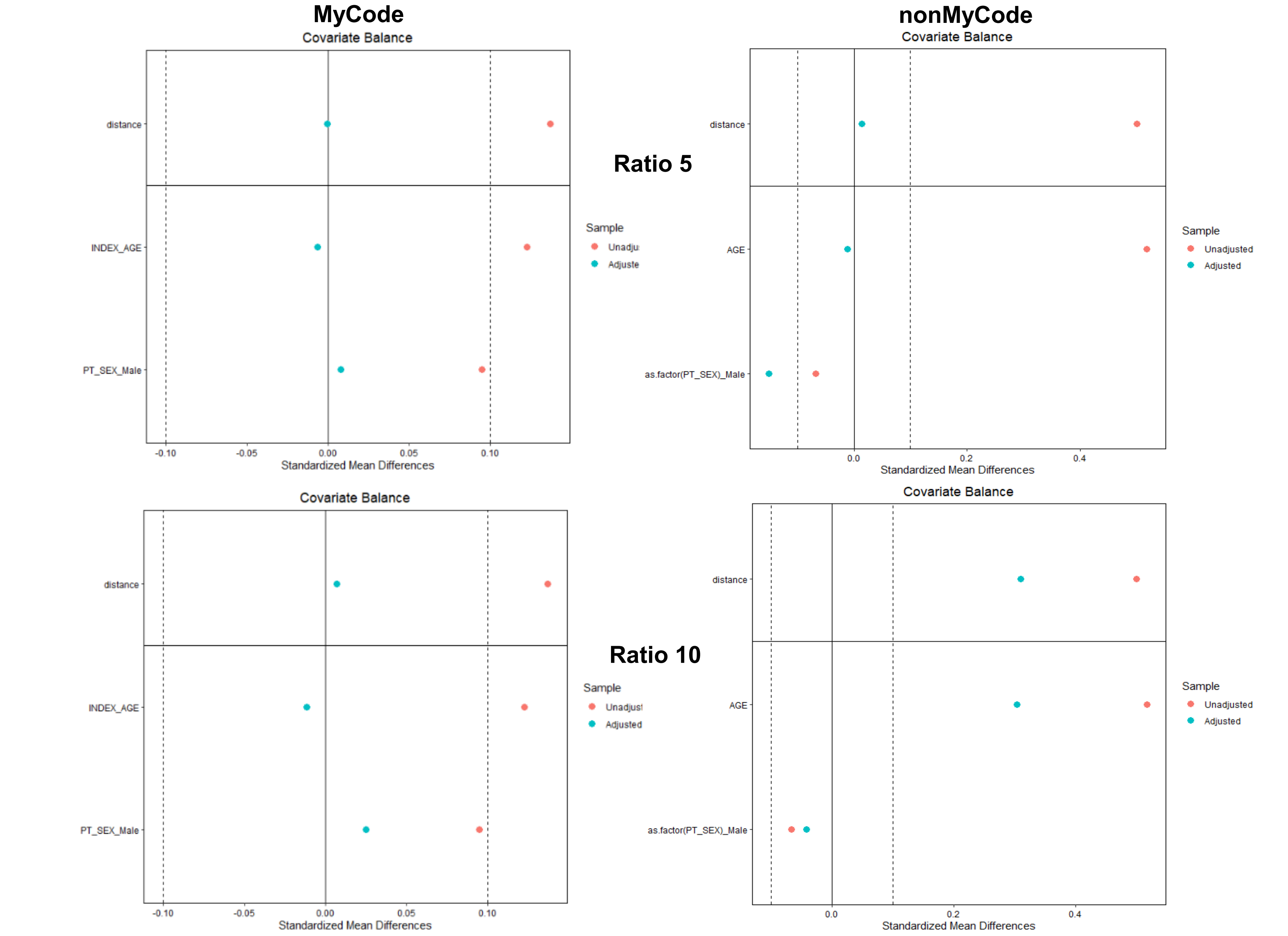

Supplement: Supplementary file 1 — Supplementary Figures. [file 41598_2023_41753_MOESM1_ESM.pdf]
