# Supplementary material for: An integrated pipeline for prediction of Clostridioides difficile infection
Source: Sci Rep. 2023 Oct 2;13:16532. doi: 10.1038/s41598-023-41753-7 (PMC10545794; doi:10.1038/s41598-023-41753-7)
Supplement: Supplementary file 3 — Supplementary Information 3. [file 41598_2023_41753_MOESM3_ESM.pdf]

Appendix:

1. Cases

1.1 Labs (gold standard)

Anyone ≥2 years of age with at least one positive inpatient or outpatient C. diff antigen or toxin test (includes positive tests for strain A, strain B, or strains A and B).

1.2. Diagnoses (silver standard)

When a patient's record includes one or more diagnosis codes for C. diff there are three ways that diagnosis code, combined with other information from the chart, may qualify the patient as a case.

Diagnoses and Medications

Anyone ≥2 years of age with the following sequence of diagnosis codes and medications:

Within a 90-day period the subject has:

1) 1+ days with an inpatient or outpatient diagnosis of C. diff, *followed by*
2) 1+ days with a dispensed medication for treating C. diff, *followed by*
3) 1+ days with an inpatient or outpatient diagnosis of C. diff.

The diagnosis codes for C. diff are:

008.45 (intestinal infection due to *C. difficile*)

The medications for treating C. diff are:

Metronidazole
Vancomycin (oral)
Fidaxomicin
Linezolid

2. Controls

2.1 Exposure to Class 1 (high risk) or Class 2 (moderate risk) Antibiotics and Hospitalization

Anyone ≥2 years of age who:

1) has no known test for C. diff (see 1.1), AND
2) has no diagnosis codes for C. diff (see 1.2.1), AND
3) has at least one hospital admission with a prior exposure to antibiotics (see next), and
4) has been exposed to a class 2 (moderate risk for C. diff) or class 1 (high risk for C. diff) antibiotic 7-62 days before the hospital admission.
5) has not had chemotherapy or evidence of bone marrow cancer for the **180** day period prior to the c-diff index date **OR** the 7 days following their index date. (These are the 'exclusionary criteria' in the Appendix A flowchart)

2.2. Exposure to Class 1 (high risk) or Class 2 (moderate risk) Antibiotics and No Mentions of C. Diff. in Progress Notes

Anyone ≥2 years of age who:

1) has no known test for C. diff (see 1.1), and Negative test for C.diff
2) has no diagnosis codes for C. diff (see 1.2.1), and
3) has been exposed to a class 2 (moderate risk for C. diff) or class 3 (high risk for C. diff) antibiotic, and
4) has continuous enrollment for 5 or more years following this antibiotic exposure, and
5) has no mentions in progress notes of C. diff (ever).
6) has not had chemotherapy or evidence of bone marrow cancer for the **180** day period prior to the c-diff index date **OR** the 7 days following their index date. (These are the 'final exclusion' in the Appendix)

3. Covariates

The following covariates will be collected for both cases and controls.

- **Basic demographic info**
  - BMI, Weight, Height (high missingness not included)
  - Genetic Gender/Ancestry (only individuals with European Ancestry included in this study)
- **Nursing home status** (not accurate in our EHR)
  - Time -90d to index date
  - Structured data on SNF residence, or
  - Text search for generic or proper names of area nursing homes, esp. within Social Work/case management notes, e.g.:
    - Generic names:
      - NH
      - NSH
      - nursing home
      - SNF
      - skilled nursing facility
      - Hospice
      - NHC

      Proper names such as:
      ▪Cumberland Manor,
      ▪Ida Culver House,
      ▪etc.
- **Chemotherapy** (Yes/No) – outside of exclusionary time range
  - Exclusionary Time: -180d to index date + 7 days
  - Chemotherapy procedures: any inpatient or outpatient chemotherapy
- **Diabetes Mellitus**
  - Time: Ever
  - either modify T2D algorithm to include all DM OR use any 2 of these: [ICD9 250.*];]DM meds including insulin]; [A1c> 6.5 OR Glucose > 200])
- **HIV**
  - Time: Ever
  - ICD9 042
- **Cancer**
  - Time: Time 0 (date of Subject's first record) to index_date + 7 days
  - ICD9 140-172.99, and 174-209.99
- **Transplant medications**
  - Time: Time 0 (date of Subject's first record) to index_date + 7 days
  - cellcept, munoloc, mycophenylate mofetil
  - tacrolimus,fk-506,fk5,k506,tacarolimus,tacrolimus hydrate,fujimycin,lcp-tacro,prograf,protopic
  - cyclosporine,ciclosporin,cyclosporin,cyclosporin a,gengraf,neoral,restasis,restasis,sandimmune,sangcya
  - azothioprine,azathioprin,azathioprine sodium,azatioprin,azamun,azanin,azasan,ccucol,imuran,imu r ek,imurel,muran
- **Corticosteroid Meds**
  - Time: -21 Days to index_date
  - Corticosteroid Medications: Cortisone (Cortisone and Cortisone Acetate), Hydrocortisone (Hydrocortisone, Hydrocortisone Sodium Phosphate, Hydrocortisone Sodium Succinate, Hydrocortisone Acetate, Hydrocortisone Cypionate), Prednisone, Prednisolone (Prednisolone and Prednisolone Sodium Phosphate), Methylprednisolone (Methylprednisolone, Methylprednisolone Sodium Succinate, Methylprednisolone Acetate), Triamcinolone (Triamcinolone, Triamcinolone Acetonide, Triamcinolone Diacetate, Triamcinolone Hexacetonide), Dexamethasone (Dexamethasone, Dexamethasone Acetate, Dexamethasone Sodium Phosphate), Betamethasone (Betamethasone, Betamethasone Sodium Phosphate, Betamethasone Acetate)
  - Exclude if strength/dose includes a "%"
  - {[Route must be (IV, IM, or PO)] OR [have a dose/strength ("MG|milligram|miligram")]} AND[Route not like intranasal|topical|inhaled]

- **Anti-TNF medications**
    - Time: -21 Days to index_date
    - infliximab
    - remicade
    - adalimumab
    - humira
    - certolizumab
    - cimzia
    - golimumab
    - simponi
    - etanercept
    - enbrel

4. Final exclusion criteria

Exclude from the set of otherwise eligible cases any subject qualifying through any of the three pathways above (i.e., 1.1 or 1.2) if any of the following conditions is met:

- The patient has any diagnosis code for bone marrow **cancer** (see Appendix C) in the **2-year** period prior to the c-diff index date **OR** the 7 days following the c-diff index date **OR**
- The patient received cancer **chemotherapy** (see Appendix D) in the **180** day period prior to the c-diff index date **OR** the 7 days following the c-diff index date

- **Antibiotic exposure** (See a list of antibitics with high and moderate risk
    - Time: -62d to -7d prior to index date
    - Classified by risk of developed CDiff following (1=high risk, 2=mod risk, 3=low risk)
- **PPI** (proton pump inhibitor) medications outside of exclusionary time
    - Exclusionary Time: -21d to index date
    - omeprazole
    - esomeprazole
    - lansoprazole
    - dexlansoprasole
    - pantoprazole
    - rabeprazole
    - Prevacid
    - Nexium,
    - Dexilant
    - Prilosec
    - Zegerid
    - Protonix
    - Aciphex
    - Vimovo
- **Inflammatory Bowel Disease**
    - ever (ICD9 555.# for Crohn's disease and 556.# for Ulcerative colitis)

High risk

- AVALOX
- avelox
- cedax
- CEFAZOLIN / CLINDAMYCIN
- cefdinir
- CEFDINIR : OMNICEF
- Cefepime
- cefixime
- cefotaxime
- cefoxitin
- cefpodoxime
- ceftazidime
- ceftriaxone
- ceftriaxone w/lidocaine
- cipro
- CIPRO / LEVOFLOXACIN
- CIPRO XR
- CIPROFLAXACIN
- ciprofloxacin
- ciprofloxacin : cipro
- CIPROFLOXACIN (CIPRO)
- CIPROFLOXIN
- CLAVULANATE (AUGMENTIN)
- cleocin
- cleocin t
- clindamycin
- clindamycin : cleocin
- clindamycin hcl
- CLINDAMYCIN HCL (CLEOCIN)
- CLINDAMYCIN PHOSPHATE
- floxin
- fortaz
- levaquin
- levaquin / ibuprofen
- levaquin leva-pak
- levofloxacin
- levofloxacin : levaquin
- maxipime
- MEFOXIN
- moxifloxacin
- omnicef
- rocephin
- tequin
- TROVAFLOXACIN
- TROVAN
- vanc / cefepime
- vanc / rocephin
- VANCOMYCIN / CEFOTAXIME
- vantin
- zithromax / rocephin
- zosyn / cipro

Moderate risk

- amox
- amoxicillin
- amoxicillin-clavulanate
- amoxil
- ampicillin
- AMPICILLIN / MEROPENEM
- AMPICILLIN SODIUM
- ampicillin-sulbactam
- ancef
- augmentin
- azactam
- azithromycin
- azithromycin : zithromax
- aztreonam
- biaxin
- BIAXIN / PENICILLIN
- BIAXIN XL
- BICILLIN
- ceclor
- cefaclor
- CEFADROXIL
- cefazolin
- CEFOTAN
- cefotetan
- CEFPROZIL
- ceftin
- cefuroxime
- cefuroxime : ceftin
- cefuroxime axetil
- cefuroxime axetil (ceftin)
- cefzil
- cephalexin
- CEPHALEXIN (KEFLEX)
- CEPHALEXIN HCL
- CEPHALOTHIN
- claforan
- clarithromycin
- CLARITHROMYCIN (GENERIC)
- CLARITHROMYCIN / AMIKACIN
- dicloxacillin
- e-mycin
- ees
- ertapenem
- ERYTHROCIN
- erythromycin
- erythromycin base
- ERYTHROMYCIN ETHYLSUCCINATE
- ERYTHROMYCIN LACTOBIONATE
- erythromycin stearate
- floxacillin
- imipenem
- imipenem / cilastatin
- imipenem-cilastatin

Moderate risk

- imipenem-cilastatin injection
- Invanz
- Keflex
- kefzol
- KETEK
- LORABID
- MERONEM
- meropenem
- MEROPENEM : MERREM
- merrem
- methicillin
- nafcillin
- oxacillin
- pen vk
- PEN-VEE K
- PEN-VK
- penicillin
- PENICILLIN G
- PENICILLIN G BENZATHINE
- PENICILLIN G POTASSIUM
- penicillin v potassium
- penicillins
- piperacillin
- piperacillin / tazobactam
- piperacillin-tazobactam
- piperacillin-tazobactam inj
- primaxin
- suprax
- ticar
- ticarcillin
- ticarcillin / clavulanate
- timentin
- trimox / amox
- ULTRACEF
- unasyn
- VANC / DORIPENEM
- vanc / zosyn
- vancomycin / doripenem
- vancomycin / ertapenem
- ZARTAN
- zinacef
- zithromax
- ZITHROMAX (ZPAK)
- zithromax z-pak
- zosyn
- zosyn / nsaids
- ZPACK
- zpak
